# Comparing the EQ-5D 3L and 5L: measurement properties and association with chronic conditions and multimorbidity in the general population

**DOI:** 10.1186/1477-7525-12-74

**Published:** 2014-05-16

**Authors:** Calypse B Agborsangaya, Markus Lahtinen, Tim Cooke, Jeffrey A Johnson

**Affiliations:** 1 Department of Public Health Sciences, 2-040 Li Ka Shing Center for Health Research and Innovation, University of Alberta, Edmonton, Alberta, T6G 2E1, Canada; 2 Health Quality Council of Alberta, Calgary, Alberta, Canada

**Keywords:** EQ-5D, Quality of life, Multimorbidity, Health related quality of life, Chronic diseases, Shannon index, Canada

## Abstract

**Background:**

Studies comparing the measurement properties of EQ-5D 3L (3L) and EQ-5D 5L (5L) are limited to specific patient populations with small sample sizes. Using a general population sample, we compared 3L and 5L in terms of their measurement properties and association with number of chronic conditions, including multimorbidity – the concurrent occurrence of two or more chronic conditions.

**Methods:**

Data were available from two consecutive cycles of a cross-sectional telephone interview survey using 3L (2010 cycle) and 5L (2012 cycle), in the general population of adults (age ≥ 18 years) in Alberta, Canada. Measurement properties were compared by determining their feasibility, ceiling effect, and discriminatory power (Shannon indices) for 3L and 5L. Linear regression models were fitted to test the associations between multimorbidity and EQ-5D index score.

**Results:**

Data were available for 4946 (2010) and 4752 (2012) survey respondents with information on HRQL. Compared to 3L, 5L showed lower ceiling effect (32.3% versus 42.1%), higher absolute discriminatory power (Shannon index, mean 0.79 versus 0.52) and higher relative discriminatory power (Shannon Evenness index, mean 0.09 versus 0.06 for 3L). Despite these differences, similar relationships of lower HRQL with greater multimorbidity were observed for the 3L (ß = −0.13, 95% CI −0.15; −0.11) and 5L (ß = −0.12, 95% CI −0.13; −0.11).

**Conclusions:**

Using a general population sample, the EQ-5D 5L showed better measurement properties than the EQ-5D 3L. Nonetheless, clinically important differences in HRQL associated with multimorbidity were similar in magnitude using both versions of EQ-5D.

## Background

For over two decades, EuroQol’s EQ-5D has been used as a generic instrument to measure and evaluate health status [1-3]. The older version, the EQ-5D 3L (3L), describes general health based on five distinct dimensions: mobility, self-care, usual activities, pain/discomfort and anxiety/depression. Each dimension has 3 Levels (indicating no problem, some or moderate problem and extreme problem). Due to its limited ability to delineate minor but important clinical differences in health status as well as the presence of a ceiling effect, the EuroQol group recently developed a new version, the EQ-5D 5L (5L) [4-7]. The 5L version differs from 3L in that for each dimension, there are 5 levels (no problem, slight problem, moderate problem, severe problem and extreme problem).

Previous research comparing the 3L and 5L versions of the EQ-5D indicate that additional levels of the 5L potentially increase discriminatory power and reduce ceiling effect among patients with chronic conditions, including chronic hepatic disease [8] or cancer patients [9]. Although few studies have compared 3L and 5L, most of them are based on patient populations or studies with relatively small sample sizes [6-9]. In one study among eighty-two participants [6], the authors compared 3L and 5L and noted a significant improvement in the discriminatory power of the 5L. More research is needed to obtain a comparative assessment of both versions of the EQ-5D in a larger sample and their applicability in the general population.

The present study aims to compare the measurement properties of 3L and 5L in terms of their feasibility, ceiling effect and discriminatory power (Shannon indices) in a general population sample. In a further step, we compared the association between multimorbidity, the concurrent occurrence of 2 or more chronic conditions, and health-related quality of life (HRQL) using both versions of the EQ-5D.

## Methods

### Study setting and population

The study sample constitutes respondents to two consecutive survey cycles of the Health Quality Council of Alberta (HQCA) Patient Experience and Satisfaction Survey [10] for 2010 and 2012. In this cross-sectional survey, adult Albertans aged 18 years or older, representative of the adult general population, self-reported their experiences and satisfaction with the quality of health services received in the past twelve months. The survey comprised of a telephone-based questionnaire that was administered by Random-Digit Dialing (RDD). In the sampling design, densely populated regions were under-sampled and sparsely populated regions were oversampled to ensure a reasonably large sample for analysis and reporting of data for each health region. This study uses combined regional samples to represent the population of Alberta. In doing so, sampling weights were applied to adjust for under- and over-sampling.

### Chronic conditions and socio-demographic factors

Respondents were asked about their health status in the past 12 months, including if they had “any of the following chronic conditions”; diabetes, chronic obstructive pulmonary disorder, asthma, hypertension, high cholesterol, sleep apnea, congestive heart failure, depression or anxiety, chronic pain, arthritis, heart disease, stroke (or related), cancer, gastro-intestinal tract and kidney diseases. Thus, this analysis includes up to fifteen chronic conditions. Respondents were also categorized as having multimorbidity if they had any two or more of these conditions. The survey respondents also gave information on their socio-demographic characteristics such as age, sex, educational attainment and household income. Unlike in 2012, the 2010 survey cycle included a skip pattern for listing chronic conditions that queried respondents to indicate if they have “any chronic condition” prior to the list of chronic conditions.

### Measures

The 2010 survey cycle included EQ-5D 3L and has been described elsewhere [3,11], whereas the 2012 cycle entailed the newer version, EQ-5D 5L. Considering that the descriptive system of the EQ-5D comprises of five domains, each with three possible levels for 3L and five levels for 5L, a combination of the characteristic levels produces 243 (3^5^) possible health states for 3L (ranging from 11111 to 33333) and 3125 (5^5^) health states for 5L (ranging from 11111 to 55555). 11111 represents the best possible health state whereas 33333 (for 3L) or 55555 (for 5L) represent the worst health states.

The EQ-5D index scores are utilities derived from the respondents profile and ranges from 0 to 1; 0 meaning death and 1 complete health [1]. Values less than 0 indicate health states worse than death. Since the EQ-5D index score is a weighted summary score of five items representing different dimensions of health, changes in the EQ-5D index score may arise from different patterns of impairment across individual dimensions. We were particularly interested in (a) the frequency of reported problems in the 3L and 5L and (b) their EQ-5D single index score. The EQ-5D index scores from 5L were derived as described elsewhere [12,13], using a Crosswalk Index Value Calculator based on US national scoring algorithms. A difference of 0.03 (3%) was considered to be clinically important [14]. Given that index scores for 5L are generated by mapping responses on to 3L, we hypothesize that significant differences in association will not be observed.

### Data analyses

#### Discriminatory power

Shannon index (H’) and Shannon Evenness index (J’) were calculated for both instruments as a measure of the discriminatory power. Shannon indices originate from the field of information theory and are typically used to measure species diversity and richness in ecological studies or information richness in the information sciences [15-17]. Some studies have shown that Shannon indices are useful in assessing the discriminatory power in the dimensions of the EQ-5D descriptive system [4,7,18,19]. H’ is calculated based on the following equation:

$$H’=-\sum_{i=1}^{L}p_{i}\log_{2}p_{i}$$

Where L stands for the total number of possible categories (levels), p_i_ = n_i_/N, the proportion of recorded observations in the *ith* level, n_i_ the observed number of responses in level *i* and *N*, the total sample size [15]. A higher H’ is attributed to more information that has been captured. To calculate H’ for an instrument (H’ _instrument_), p_i_ is generated for the total number of responses for a given level across all dimensions as a proportion of total responses to the entire instrument [4]. Similarly, a higher H’ _instrument_ indicates that more information is captured by that instrument. Shannon Evenness Index, J’ , is a complementary index that reflects the evenness of a distribution and is defined as: J’ = H’/ H’ _max_, where:

$$H{’}_{\text{max}}={\mathrm{Log}}_{2}L$$

Both indices are descriptive measures of the discriminatory ability of an instrument and are needed for useful interpretation of the measurement power of a scale. We expect that the H’ for 5L should be higher than for 3L, and that J’ should be about the same or slightly higher for 5L, indicating the usefulness of the extra levels in the 5L.

#### Feasibility

We tested feasibility by computing the percentage of respondents with missing responses. That is, we calculated the percentage of respondents not answering each dimension. The percentages were then compared for both the 3L and 5L.

#### Ceiling effect

The ceiling of 3L and 5L were defined as the proportion of respondents that scored no problem on all five dimensions (persons with 11111 score). Based on the assumption that the majority of respondents would report at least slight/some problem on at least one of the five dimensions, we hypothesis that the ceiling effect will be lower for 5L compared to 3L version.

### Statistical methods

Descriptive statistics were performed to determine the sample characteristics for each survey cycle. We presented the proportion of reported problems in all five dimensions for each cycle (without merging) to provide information on the advantage of the extra levels in each dimension. Multivariable linear regression models were then fitted to study associations between chronic conditions and EQ-5D index scores. Three separate models were fitted for 1) specific chronic conditions, 2) for number of chronic conditions and 3) for presence of multimorbidity as a binary categorical variable. The multivariable models were adjusted for respondents’ socio-demographic characteristics such as age, sex, and household income. Because educational level tends to correlate with income, we determined a priori not to adjust for educational level to avoid multicollinearity. A 2-sided P < 0.05 was considered statistically significant. All analyses were undertaken using STATA version 11 (StataCorp LP. 2009). The Health Research Ethics Board (HREB) at the University of Alberta approved the data collection protocols and survey instruments.

## Results

### Socio-demographic characteristics of respondents

4946 (98.7%) and 4752 (98.9%) respondents reported data on the EQ-5D in 2010 and 2012 survey cycles, respectively. Respondents’ socio-demographic characteristics were comparable in both surveys (Table 1). 52.3% were female and the mean age was 46.6 (16.5) years for the 2010 cycle, compared to 55.7% female and mean age of 47.7 (SD, 17.1) years for the 2012 survey. The proportions of respondents reporting five or more chronic conditions were 159 (3.2%) and 301 (6.3%) for the 2010 and 2012 survey cycles, respectively. The mean number of chronic conditions was 0.7 (SD = 1.4) for the 2010 survey cycle and 1.3 (SD = 1.7) for the 2012 survey cycle.

**Table 1:** **Socio**-**demographic characteristics of the survey respondents**

	**2010 Survey (N = 4946)**	**2012 Survey (N = 4752)**
Mean age (SD), years	46.6 (16.5) years	47.7 (17.1) years
Sex		
Females (%)	2585 (52.3)	2647 (55.7)
Age (%)		
18- 28	753 (15.2)	724 (15.2)
29-38	960 (19.4)	884 (18.6)
39-48	1035 (20.9)	797 (16.8)
49-58	987 (20.0)	971 (20.4)
59-68	667 (13.5)	799 (16.8)
≥ 69	544 (11.0)	577 (12.1)
Education (%)		
Secondary or less	1747 (35.5)	1634 (34.5)
Post-secondary/College	2008 (40.8)	1896 (40.0)
University degree	1170 (23.8)	1207 (25.5)
Household income (CAD), %		
< 30,000	579 (13.5)	624 (15.1)
30,000 – 59,999	1019 (23.8)	926 (22.4)
60,000 – 99,999	1230 (28.7)	1097 (26.5)
≥ 100,000	1454 (34.0)	1496 (36.1)
Number of chronic conditions		
0	3281 (66.7)	2277 (47.9)
1	751 (15.3)	878 (18.5)
2	387 (7.9)	644 (13.6)
3	210 (4.3)	388 (8.2)
4	134 (2.7)	264 (5.6)
5+	159 (3.2)	301 (6.3)

### EQ-5D profile and morbidity status

The HRQL profile of persons with multimorbidity is summarized in Table 2. Among persons with multimorbidity, the proportion of respondents reporting *slight problems* was higher for each dimension of 3L compared to 5L. For instance, the proportion of persons reporting slight problems with mobility was 45.2% for 3L compared to 25.0% for 5L. The same was true for *usual activity* (69% for 3L versus 23.0% for 5L). Pain/discomfort appeared to be most common among persons with multimorbidity.

**Table 2:** **EQ**-**5D profile** (**3L and 5L**) **of the survey respondents with multimorbidity**

**Multimorbidity (3L)**			**Multimorbidity (5L)**		
**Dimension**	**n**	**%**	**n**	**%**	**Dimensions**
**Mobility**					**Mobility**
No problems	487	54.0	823	50.7	No problems
Slight problems	408	45.2	406	25.0	Slight problems
Extreme problems	7	0.8	276	17.0	Moderate problems
			104	6.4	Severe problems
			13	0.8	Unable to walk about
**Selfcare**					**Selfcare**
No problems	813	89.7	1438	88.4	No problems
Slight problems	91	10.0	113	7.0	Slight problems
Extreme problems	2	0.2	60	3.7	Moderate problems
			9	0.6	Severe problems
			6	0.4	Unable to wash or dress
**Usual activities**					**Usual activities**
No problems	498	55.0	868	53.6	No problems
Slight problems	380	41.9	372	23.0	Slight problems
Extreme problems	28	3.1	269	16.6	Moderate problems
			82	5.1	Severe problems
			28	1.7	Unable to do usual activity
**Pain/Discomfort**					**Pain/Discomfort**
No problems	170	18.8	280	17.3	No problems
Slight problems	627	69.2	586	36.2	Slight problems
Extreme problems	109	12.0	535	33.1	Moderate problems
			174	10.8	Severe problems
			43	2.7	Extreme pain
**Anxiety/Depression**					**Anxiety/Depression**
No problems	534	59.5	868	53.8	No problems
Slight problems	314	35.0	391	24.2	Slight problems
Extreme problems	49	5.5	260	16.1	Moderate problems
			66	4.1	Severe problems
			29	1.9	Extreme problems

### Specific chronic conditions and EQ-5D index score

The mean (SD) index score was 0.87 (SD 0.15) for 3L and 0.86 (SD, 0.14) for 5L. The associations between specific chronic conditions and HRQL are presented in Table 3. In the multivariate analysis, all chronic conditions were associated with clinically important differences in EQ-5D index score using both 3L and 5L. Overall, the associations tended to be similar, albeit, coefficients were larger for 3L compared to 5L for anxiety or depression, chronic pain, arthritis and sleep apnea (Table 3), in some cases larger than the minimally important difference.

**Table 3:** Associations between specific chronic conditions and EQ index score

**Chronic conditions**	**3L index score Coef. (95% CI)**^**1**^	**5L index score Coef. (95% CI)**^**2**^
Diabetes	−0.05 (−0.08, −0.02)	−0.04 (−0.06, −0.02)
COPD	−0.10 (−0.16, −0.04)	−0.08 (−0.11, −0.05)
Asthma	−0.06 (−0.09, −0.03)	−0.06 (−0.07, −0.04)
High blood pressure	−0.06 (−0.08, −0.04)	−0.06 (−0.07, −0.04)
High cholesterol	−0.05 (−0.08, −0.03)	−0.04 (−0.06, −0.03)
Sleep apnea	−0.13 (−0.17, −0.09)	−0.09 (−0.11, −0.06)
Congestive heart failure	−0.12 (−0.21, −0.03)	−0.12 (−0.19, −0.05)
Depression or anxiety	−0.19 (−0.21, −0.16)	−0.14 (−0.15, −0.13)
Chronic pain	−0.19 (−0.21, −0.17)	−0.17 (−0.18, −0.15)
Arthritis	−0.12 (−0.14, −0.10)	−0.10 (−0.12, −0.09)
Heart disease	−0.06 (−0.10, −0.02)	−0.06 (−0.09, −0.03)
Stroke (or related)	−0.11 (−0.19, −0.02)	−0.12 (−0.17, −0.06)
Cancer	−0.06 (−0.11, −0.01)	−0.05 (−0.08, −0.02)
Kidney disease	−0.07 (−0.20, 0.05)	−0.14 (−0.19, −0.09)
Bowel disorders	−0.09 (−0.13, −0.06)	−0.10 (−0.13, −0.08)

^1^EQ-5D 3L from 2010 Survey and ^2^EQ-5D 5L from 2012 surveys, adjusted for age, sex and household income.

### Multimorbidity and EQ-5D index score

The associations between number of chronic conditions, including multimorbidity, and EQ-5D index score is presented in Table 4. In the multivariable analysis, having a chronic condition was associated with clinically important differences in EQ-5D index score using both 3L and 5L. The difference in EQ-5D index score tended to increase with increasing number of chronic conditions, being highest for persons with five or more conditions ((ß = −0.23 (95% CI −0.27, −0.19) for 3L and (ß = −0.22 (95% CI −0.23, −0.20) for 5L). Multimorbidity was associated with clinically important differences in EQ-5D index score {(ß = −0.13 (95% CI −0.15, −0.11) for 3L and ß = −0.12 (95% CI −0.13, −0.11) for 5L).

**Table 4:** Associations between multimorbidity and EQ index score

**Number of chronic conditions**	**EQ-5D 3L index score Coef. (95% CI)**^**1**^	**EQ-5D 5L index score Coef. (95% CI)**^**2**^
0	Reference	Reference
1	−0.07 (−0.09, −0.06)	−0.06 (−0.07, −0.05)
2	−0.12 (−0.14, −0.10)	−0.10 (−0.12, −0.09)
3	−0.14 (−0.17, −0.11)	−0.12 (−0.14, −0.11)
4	−0.16 (−0.20, −0.11)	−0.18 (−0.21, −0.15)
≥ 5	−0.23 (−0.27, −0.19)	−0.22 (−0.23, −0.20)
**Multimorbidity**		
No	Reference	Reference
Yes	−0.13 (−0.15, −0.11)	−0.12 (−0.13, −0.11)

Difference in EQ-5D index score for the ^1^2010 and ^2^2012 Surveys of the HQCA; Adjusted (age, sex and household income).

### Comparing measurement properties of EQ-5D 3L and EQ-5D 5L

Missing values ranged from 0.1% for Self Care to 0.8% for Anxiety/Depression for the 3L version and 0.1% for Self Care to 0.6% for Anxiety/Depression for the 5L version. The proportion of respondents with at least one missing value in all dimensions was 1.3% for the 3L version and 1.1% for the 5L version, indicating good feasibility for both versions of the instrument.

There were a total of 384 unique health states observed using the 5L (12.2% of 3125 possible) and 116 (47.7% of 243 possible) using the 3L version. A ceiling effect was observed among a greater proportion of respondents with the 3L, 2082/4946 (43.1%) compared to 5L, 1536/4752 (32.3%), with an absolute difference of 9.8%. Among the most common chronic conditions, the highest difference was noted for high blood pressure (20.6% versus 14.3%) and high cholesterol (20.1% versus 14.8%) and similar for arthritis (6.9% vs. 6.8%) and anxiety/depression (3.0% for both).

**Figure 1:**
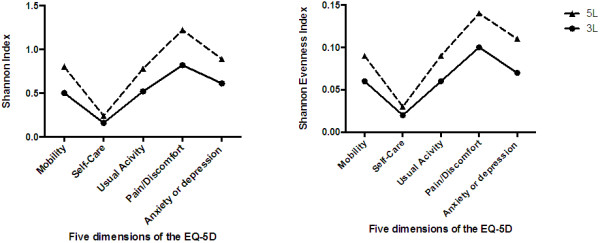
Shannon index (H’) and Shannon Evenness index (J’) values for the 3L and 5L.

EQ-5D instrument-dependent discriminatory power is represented by Shannon indices in Figure 1. Absolute discriminatory power (Shannon Index) was substantially higher in information richness in the 5L classification system for all dimensions (H’ _5L_/H’ _3L_): Mobility (0.80/0.50); Self-Care (0.24/0.16); Usual Activity (0.79/0.52); Pain/Discomfort (1.22/0.82), Anxiety/Depression (0.89/0.61) and overall (0.87/0.59). Relative discriminatory power (Shannon Evenness index) that emphasizes the evenness of spread of responses across all levels by adjusting for the number of levels per instrument was slightly higher for all dimensions of 5L (J’ _5L_/J’ _3L_): Mobility (0.09/0.06); Self-Care (0.03/0.02); Usual Activity (0.09/0.06); Pain/Discomfort (0.14/0.10); Anxiety/Depression (0.11/0.07) and overall (0.09/0.06). The absolute discriminatory power was higher on average with the 5L (mean 0.79 versus 0.52 for 3L), and the relative discriminatory power was also reasonably higher for the 5L (mean 0.09 versus 0.06 for 3L), confirming our hypothesis.

## Discussion

The present study compares the measurement properties of EuroQol’s EQ-5D 3L and the newer 5L in terms of their feasibility, ceiling effect and discriminatory power (Shannon indices) as well as the association between clinical characteristics and EQ index score. In our comparison of 3L and 5L, both were comparable in terms of feasibility, but 5L had lower ceiling effect and higher discriminatory power. Overall, multimorbidity was associated with clinically important reduction in index scores using both versions of the instrument, although it was notable that the decrements associated with specific conditions were different between the 3L and 5L version.

This population-based study utilizes large samples from two consecutive survey cycles that are representative of the general population. Chronic pain was associated with the highest clinically important difference in HRQL using both versions of the EQ-5D instrument, consistent with previous findings [3,20,21]. However, the magnitude of this difference tended to be larger using the 3L compared to the 5L for some chronic conditions. The difference for individual conditions such as chronic pain and anxiety or depression may be due to different weights for specific dimensions associated with symptoms of these chronic conditions. That is, these conditions have the biggest weight on level 3 of the 3L. Likely related to the symptom of pain, the score difference associated with arthritis was also larger for the 3L than 5L.

On the other hand, the associations between chronic conditions and index scores were similar for most chronic conditions, including multimorbidity using both versions of the EQ-5D. This similarity may be due to the fact that health profiles of 5L are mapped unto the 3L to derive its index score using an interim scoring “*EQ*-*5D 5L Crosswalk Index Value Calculator*” [13]. That is, the utilities for 5L are derived by first cross-matching its profiles to those of 3L. It is therefore expected that studied associations using index scores from both versions will not be significantly different. On the other hand, similar findings may in fact indicate the lack of difference in 3L and 5L using their derived utility scores. As a unique scoring system is being awaited for 5L, further studies will be required to show if differences occur in association between clinical characteristics and HRQL for both versions of the instrument.

The observation that 5L had lower ceiling effect than the 3L has been reported in other studies using patient populations [7-9]. The finding supports the original intent of increasing the levels in 5L, to capture differences in health states that are otherwise not captured by 3L. Moreover, the 5L version of the EQ-5D descriptive system had a higher absolute discriminatory power than the 3L version in all five dimensions. Also, the relative discriminatory power (Evenness index) was slightly better in the 5L than the 3L version. This measure indicates the evenness of spread of responses across levels of the instrument by adjusting for the number of levels. Thus, higher evenness scores in all 5L dimensions indicate that the extra levels in the descriptive system were used efficiently. Our study findings indicate that the measurement properties of 5L are better than 3L in a general population sample. Further longitudinal analysis is needed to compare the sensitivity of both versions of the EQ-5D and their ability to detect change over time.

This study has a few limitations that are worth mentioning. Although the source population was the same in both survey cycles, the study samples for instrument comparison are not the same. The average number of chronic conditions was higher in the 2012 compared to 2010 survey cycle. This may be due to inherent differences in the study samples or under-reporting in the 2010 cycle, especially because of a skip pattern in the order of questions for identifying chronic conditions in that cycle. It is unclear to what extent this difference has on the comparison. It is unlikely to affect our comparison of specific conditions that have a higher prevalence, but may limit comparisons of total chronic conditions in the population. Furthermore, the results are consistent with findings from previous studies that are based on the same samples to validate both versions of the instrument [6-8]. Respondents’ chronic conditions were self-reported. Because chronic conditions can be quite subtle, it may be confusing to differentiate between symptoms or minor ailments with more severe disease states. Moreover, some conditions such as chronic pain are subjective and may be difficult to define without assistance from a clinician or the use of standardized scales. The severity of chronic conditions, an important predictor of HRQL [2], was not accounted for in the present study. On the other hand, a unique property of our study is the large sample size derived from the general population, heightening the external validity of our study findings. Also, the study captured common chronic conditions in the general population, including the core chronic conditions recommended for inclusion in multimorbidity indices [22].

## Conclusions

In this study, we found that the EQ-5D 5L showed better measurement properties, with lower ceiling effect and better discriminatory power than the 3L version. Furthermore, while the association between overall multimorbidity and index scores is comparable using both versions of the EQ-5D, the different versions suggest notable differences in HRQL burden for individual chronic conditions.

## Competing interests

The authors declare that they have no competing interests.

## Authors’ contributions

ACB: conception and design, statistical analysis and interpretation of data, drafting manuscript, revision of manuscript ML: data acquisition, survey instrument and design, critical revision of manuscript TC: data acquisition, survey instrument and design, critical revision of manuscript JAJ: conception and design, data acquisition and interpretation of data, critical revision of manuscript. All authors read and approved the final manuscript.
